# Exploring uranium bioaccumulation in the brown alga Ascophyllum nodosum: insights from multi-scale spectroscopy and imaging

**DOI:** 10.1038/s41598-023-49293-w

**Published:** 2024-01-10

**Authors:** Micol Zerbini, Pier Lorenzo Solari, Francois Orange, Aurélie Jeanson, Catherine Leblanc, Myriam Gomari, Christophe Den Auwer, Maria Rosa Beccia

**Affiliations:** 1 Institut de Chimie de Nice, UMR 7272, Université Côte d’Azur, CNRS, 06108 Nice, France; 2 Synchrotron SOLEIL, L’Orme des Merisiers, Départementale 128, 91190 Saint-Aubin, France; 3 Université Côte d’Azur, Centre Commun de Microscopie Appliquée, 06108 Nice, France; 4 Station Biologique de Roscoff, UMR 8227, Sorbonne Université, CNRS, 29680 Roscoff, France

**Keywords:** Marine chemistry, Marine chemistry

## Abstract

Legacy radioactive waste can be defined as the radioactive waste produced during the infancy of the civil nuclear industry’s development in the mid-20th Century, a time when, unfortunately, waste storage and treatment were not well planned. The marine environment is one of the environmental compartments worth studying in this regard because of legacy waste in specific locations of the seabed. Comprising nearly 70% of the earth’s service, the oceans are the largest and indeed the final destination for contaminated fresh waters. For this reason, long-term studies of the accumulation biochemical mechanisms of metallic radionuclides in the marine ecosystem are required. In this context the brown algal compartment may be ecologically relevant because of forming large and dense algal beds in coastal areas and potential important biomass for contamination. This report presents the first step in the investigation of uranium (U, an element used in the nuclear cycle) bioaccumulation in the brown alga *Ascophyllum nodosum* using a multi-scale spectroscopic and imaging approach. Contamination of *A. nodosum* specimens in closed aquaria at 13 °C was performed with a defined quantity of U(VI) (10^–5^ M). The living algal uptake was quantified by ICP-MS and a localization study in the various algal compartments was carried out by combining electronic microscopy imaging (SEM), X-ray Absorption spectroscopy (XAS) and micro X-ray Florescence (μ-XRF). Data indicate that the brown alga is able to concentrate U(VI) by an active bioaccumulation mechanism, reaching an equilibrium state after 200 h of daily contamination. A comparison between living organisms and dry biomass confirms a stress-response process in the former, with an average bioaccumulation factor (BAF) of 10 ± 2 for living specimens (90% lower compared to dry biomass, 142 ± 5). Also, these results open new perspectives for a potential use of *A. nodosum* dry biomass as uranium biosorbent. The different partial BAFs (bioaccumulation factors) range from 3 (for thallus) to 49 (for receptacles) leading to a compartmentalization of uranium within the seaweed. This reveals a higher accumulation capacity in the receptacles, the algal reproductive parts. SEM images highlight the different tissue distributions among the compartments with a superficial absorption in the thallus and lateral branches and several hotspots in the oospheres of the female individuals. A preliminary speciation XAS analysis identified a distinct U speciation in the gametes-containing receptacles as a pseudo-autunite phosphate phase. Similarly, XAS measurements on the lateral branches (XANES) were not conclusive with regards to the occurrence of an alginate-U complex in these tissues. Nonetheless, the hypothesis that alginate may play a role in the speciation of U in the algal thallus tissues is still under consideration.

## Introduction

One of the main challenges of the nuclear fuel cycle is waste management and the associated risk of release of metallic radionuclides in the environment^1^. Such risk is amplified by the dual (chemical and radiological) toxicity of these elements, which makes metallic radionuclides more hazardous than other heavy metals therefore making nuclear waste management a critical societal issue.

It has been documented that as a legacy of nuclear activities from the twentieth century, limited amounts of low-level radioactive waste coming from research, medicine and nuclear industry activities have been discarded in the Atlantic and Pacific Oceans^2^. The marine environment is one of the environmental compartments worth studying in this regard because of legacy waste in specific locations of the seabed. Oceans and seas also represent the final and largest (70% of Earth surface) repository for contaminated waters from rivers. Unpackaged solid and liquid waste were disposed of containing beta-emitter elements such as strontium-90, caesium-137 and cobalt-60 and low quantities of alpha-emitting nuclides with plutonium and americium^2^. In particular, between the 1950 and 1990 around 200,000 barrels of radioactive waste were dumped into the Atlantic Ocean. One of the last examples is the discard operation performed in 1982 at a site about 550 km off the European continental shelf in the Northeast Atlantic^3^. Moreover, the passing of a bill from the Japanese cabinet in 2021 allowing the discharge of the Fukushima treated nuclear water into the Pacific Ocean^4^, the study of the impact on the marine environment and more specifically on the whole trophic chain will again become a global concern^5^. For this reason, the potential dispersion of metallic radionuclides in the marine ecosystem and their toxic effects on marine organisms are of interest. In addition, seawater decontamination is extremely challenging because of strong dilution factors, streams and high salinity. As a result, marine organisms are likely to be exposed to nuclear contamination, even at low concentrations, which can affect their biological processes in the short or in the long term.

Among marine organisms, macroalgae play a paramount role in coastal marine ecosystems: they are at the basis of several trophic chains and economical resources, provide habitat for a wide range of marine organisms. Marine macroalgae are currently among the most efficient biosorption materials^6,7^. Their ability to accumulate large amounts of metals from the surrounding seawater^8^ and their broad distribution in diverse environmental conditions make them suitable biomarkers of heavy metal pollution^6,9–11^. Therefore, assessing the impact of metallic radionuclides from nuclear wastes on macroalgae is an important question, although relatively unexplored, as it may have important consequences on the marine ecosystems and in fine on the global environment^12^.

Data already reported in the literature have shown the ability of several macroalgal and microalgal species to accumulate considerable amounts of radionuclides and have determined the corresponding concentration factors^13,14^. Different approaches have been reported to explain the mechanism involved in the removal process carried out using algae as a biosorbent^15,16^ for several trace metal elements such as cadmium^17^, chromium^18^, lead^19^ and zinc^20^, but mainly on fresh water microalgal species. Such processes can be grouped into two principal categories: a metabolism-dependent bioaccumulation and a metabolic-independent biosorption. In the first case, there is an active process in the algae that incorporates the metal in the cells^21^. The biosorption mechanism, instead, is characterized by physico-chemical phenomena such as ionic exchange, surface precipitation and adsorption, not linked to the metabolic activity of the algae^22^.

Many brown macroalgae biomasses have demonstrated potential as biosorbent materials for removing radionuclides, especially uranium ions^23–25^ from aqueous solutions.

Among the metallic radioelements of interest, uranium (U) is the most widely used for nuclear energy production, at the basis of the nuclear fuel cycle. Often present in natural aerobic environments under the uranyl oxocationic form UO_2_^2+^, it is the radioelement with highest natural concentration in seawater (10^–8^ M^26^). Its main species in seawater were previously shown to be the three carbonated complexes Ca_2_UO_2_(CO_3_)_3_ and CaUO_2_(CO_3_)_3_^2−^^27,28^. Although it is a relatively weak radiotoxic, U exhibits a significant chemical toxicity, as it is able to interact with biological targets, resulting in heavy metal poisoning. Such toxicity, together with U behavior in environmental and physiological media, strongly depends on its chemical speciation and the molecular mechanisms of its accumulation by living organisms. In this framework, few studies have explored U interaction with the marine biosphere^29–32^, which still remains a key step to assess its possible biomagnification through the food webs, evaluate its global environmental impact and, consequently, define effective strategies to improve the sustainability of nuclear waste management. Lastly, U under its uranyl form is a good model, easy to manipulate, for heavier elements like plutonyl and neptunyl that may also occur under this oxocationic form in case of release in seawater^33^.

In this report we explore the accumulation mechanisms and provide initial data on the in vivo speciation of U in a marine macroalga. The brown macroalga *Ascophyllum nodosum* was chosen as a model organism due to its wide geographical presence in the Atlantic coastal shores^34^ and its high tolerance to chemical toxicity, with a well-known ability to accumulate metals^35–38^. However, little is known about the in vivo accumulation mechanisms of metal ions in *A. nodosum,* both at macroscopic and molecular level. More specifically, no literature data have been found regarding the radionuclide contamination effect on this macroalga.

The total U accumulation by *A. nodosum* has been investigated, together with its biodistribution inside different algal compartments: receptacles, lateral branches and central part of the thallus^34^. Along the thallus, these three parts represent distinct algal tissues with different physiological and developing stages. The central branch is considered the oldest, characterized by a cell wall with more than two layers, in this perennial alga. The meristematic zone, i.e. responsible of the growth, is located in the apex of lateral branches, which are therefore made of younger tissue with fewer layers in the cell wall The oval receptacles are the reproductive organs of this dioecious alga, housing fertile oospheres for female or antherozoids for male individuals. If *A. nodosum* has been extensively characterized in term of global chemical composition^34^, specific tissue data are still rare. Only in a recent review, it has been reported that the intercellular space of the medulla in the vegetative part of the thallus is predominantly occupied by insoluble aqueous substances, such as alginic acid, while the medullary regions of receptacles may contain a soluble complex substance, including alginic acid and also fucoidans, inorganic salts and probably phenols^34^.

The relative contribution of active metabolic accumulation and passive sorption was assessed by comparing U uptake data by live and dry algal specimens. Finally, using a combination of micro–X-ray Fluorescence Spectroscopy (μ-XRF), Extended X-ray Absorption Fine Structure (EXAFS) and Scanning Electron Microscopy (SEM) U localization and speciation were investigated in the different parts and tissues of *A. nodosum*.

## Materials and methods

### Biological material and seawater

The *A. nodosum* individuals used in this study were collected in the inter-tidal zone in front of the Station Biologique of Roscoff (SBR) (48° 43′ 42.4" N, 3° 59′ 26.9" W), Brittany (France). Fresh algae have been stored in a running seawater aquarium, under natural light up to maximum 2 days before being shipped alive to the laboratory. Then the algae have been kept alive in an aquarium (50 cm × 30 cm × 35 cm) filled with seawater and equipped with aerating filters.

The seawater temperature inside the aquarium has been maintained between 13°C and 15°C using an external water-cooling system to maintain the algae in constant temperature conditions.

The seawater used for the contamination experiments and algal storage has been collected at 5 m of depth in front of the Laboratoire Océanographique at Villefranche-sur-mer (UMR 7093, 43° 41′ 44.4" N, 7°18′28.0"E), Mediterranean Sea (France). The seawater has been passed through a Filter Spun 9″7/8 (Crystal filter, PP-01–978) at 1 μm to eliminate particles and to remove part of microorganisms. Seawater pH has been maintained at 8.1 during all the experiments, to avoid any change in the uptake process and the chemical speciation.

### Contamination and releasing experiments

Natural uranium (^Nat^U) was used for all experiments, that is a mixture of its three isotopes at the relative concentrations found in nature: 99.27% of U-238, 0.72% of U-235, and 0.0056% of U-234 by mass. One stock solution of ^Nat^U at [U] = 0.5 M was prepared by dissolving 1.8 g of uranyl nitrate hexahydrate (UO_2_(NO_3_)·6H_2_O,analytical grade) in diluted high purity nitric acid (0.1 M). The solution was prepared at room temperature, stirred, and kept at 4 °C in the fridge until its use for contamination experiments.

In all contamination experiments several individuals of *A. nodosum* with an average length of 15 cm and wet weight of 20 g have been selected and put in separated aquaria (20 cm × 20 cm × 25 cm) containing 4 L of seawater, previously cooled at 13 °C. Three kinds of contamination were carried out:

- Living-algae contamination cycles were carried out after one day of acclimation inside the aquaria by spiking known amounts of uranyl stock solution at regular time intervals, (80 μL every 24 h, for a total exposure period of 10 days, [U] = 1.0 × 10^−6^ M at each spike, uranium final concentration [U] = 1.0 × 10^−5^ M). In order to maintain a constant pH of the seawater inside the aquarium, 1 mL of sodium hydroxide at 0.1 M was introduced right before each spike. The concentration of U in seawater was monitored daily by collecting seawater samples in three different parts of the aquarium. The amount of U inside the algal compartments (receptacles, lateral branches and thallus) was also quantified at the end of the contamination period.
- Dry-algae uptaking studies were performed with individuals previously dried in a heater (Memmert, UNI 60, Federal Republic of Germany) at 60 °C for 4 days. Before contamination, to avoid the rapid passive sorption due to the natural hydration process of dried algal tissues, the desiccated algae were rehydrated with seawater for 1 night at room temperature.
  The contamination of dry material was carried out by reaching the desired concentration of U in seawater in one single spike of 800 μL ([U] = 1.5 × 10^−5^ M), monitoring the rate of uptake for 10 days and the final bioaccumulation factor (BAF).
- Uranium depuration by *A. nodosum* was explored in two different aquaria where the algae had been previously contaminated with one single spike of 200 μL for Aq1 and 400 μL for Aq2 (Aq.1 [U] = 2.5 × 10^–5^ M and Aq.2 [U] = 5.0 × 10^–5^ M) and then transferred into new aquaria containing 4L of fresh, non-contaminated seawater. Depuration was quantified by the daily measuring of the U concentration in seawater for a total period of 12 days.

A control experiment has been performed by contaminating an aquarium without any alga. The uranium concentration in the control experiment was measured by ICP-MS on daily collected samples, no U precipitation nor significant adsorption on the aquarium vessel has been observed and confirmed the final concentration of uranium.

The amount of uranium in seawater and algal samples was quantified by ICP- MS (inductively coupled plasma mass spectrometry).

### Determination of uranium concentration

Seawater samples were diluted 100 times and then directly analyzed by ICP-MS for U quantification.

After 10 days of contamination, algal individuals were removed from the aquaria, rinsed with distilled water to remove U potentially sorbed on the surface. The rinsing water was analyzed by ICP-MS to evaluate the amount U possibly present. Algal individuals were freeze-dried at – 20 °C (Multi-Dry, FTS® System, Inc. Ontario, Canada) until constant weight. Samples were then ground in a hermetic blade mill-stainless steel (Hachoir Caso 1749, Germany), homogenized and 0.15 g of dry material were digested in 10 mL of 68% HNO_3_ (Suprapure grade. WVR, Canada) in a microwave (ETHOSTM EASY, Milestone Srl, Italy)^39^. Digested samples were then transferred into metal-free PP (polypropylene) tubes and 2 mL of H_2_O_2_ (Suprapure grade. WVR, France) were added. Tubes were heated at 100 °C for 4 h to evaporate all the reagents. The final residues were taken up with 10 mL of HNO_3_ 2% and transferred to a tube suitable for ICP-MS analysis.

**Figure 1:**
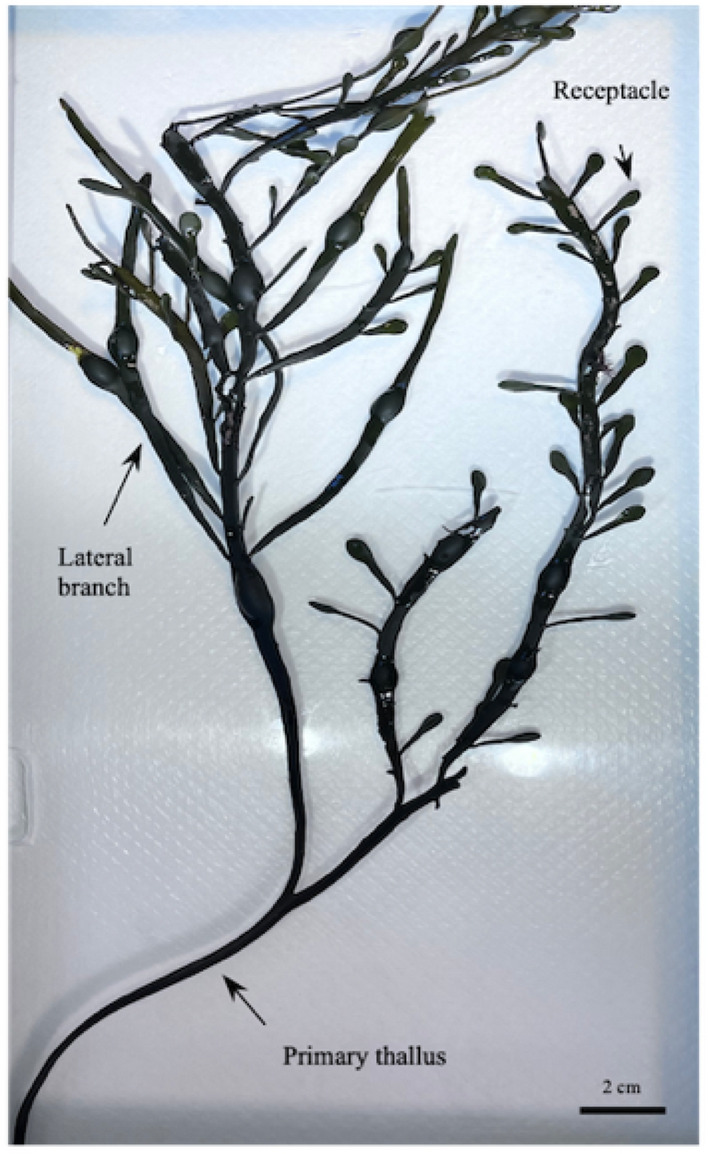
An example of *A. nodosum* individual selected for the contamination test. The image highlights the three main compartments categorized as the main axis of the alga, the thallus, that divides in dichotomous and lateral branches, containing air bladders. The apical part of the alga presents several groups of receptacles with an ovoid shape which mature in male or female conceptacles in spring for the blooming reproduction^34^.

To study the U compartmentalization inside algae, some specimens have been divided into their main compartments: receptacles, lateral branches, and central thallus (Fig. 1). Each compartment was analyzed separately and in triplicate.

All analyses were carried out on a PerkinElmer ELAN DRC-e ICP-MS (Shelton, USA) using a solution of 5 μg·L^−1^ of Thulium as internal standard (PlasmaCAL, SCP Science, Canada). ICP-MS analysis was conducted following the calibration line assessment. To prevent any potential cross-contamination, a blank was meticulously inserted after every three samples. Furthermore, at regular intervals of every six samples, either a standard from the calibration line or a quality control sample (previously prepared with a known quantity of the U standard solution) was analyzed to quantify instrumental deviations during measurements.

Calibration curves (0.1–50 µg·L^−1^) has been prepared with uranium standard (Uranium Pure Plus Standard, 2% HNO_3_, PerkinElmer) using two different methods: external for the analysis of the digested-biological samples and a matrix matching for the seawater.

The instrument response was periodically checked with a multistandard solution (PlasmaCAL, SCP Science, Canada).

U concentration (mg·Kg^−1^) in algae specimens over time was calculated as the difference between the amount of spiked U and that of uranium measured in daily collected seawater samples (see Fig. S1 of SI).

The Bioaccumulation factor (BAF), was evaluated as a ratio between U concentration in algae samples and in seawater, according to^9^ (Eq. (1)):

1 $${\text{BAF}}={C}_{t}/{C}_{S}$$

where Ct is the concentration of U in algae tissues, Cs is the concentration of U in seawater at the end of the contamination period. BAF is dimensionless.

### Alginate extraction

Alginate was extracted from *A. nodosum* individuals by adapting the Sellimi et al. procedure^40^. Freeze-dried and milled biomass was soaked in EtOH (70% v/v) for one night, to eliminate pigments and fatty acids^41^. The filtered solid was then immersed in a 0.2 M HCl (Merk, Germany) solution and left for 24 h, to obtain the insoluble alginic acid^42^. After this time, the sample was washed with distilled water and extracted with 2% Na_2_CO_3_ for 5 h at 60 °C under magnetic agitation, to solubilize the alginate in its sodium salt form. The supernatant was collected by centrifugation (15,000 *g*, 30 min and 15 °C). Alginate was isolated by precipitation in EtOH (3:1 v:v, 90%, − 18 °C) and washed twice in acetone. To remove the carbonate impurities the extract recovered was then suspended in distilled water, acidified with HCl (pH < 3) to reobtain the alginic acid. The precipitate, separated by centrifuge (4500 *g*, 10 min and 20 °C), was suspended again in distilled water and basified with NaOH (pH > 8) (Sigma Aldrich, Germany). Finally, the purified alginate was precipitated in absolute EtOH^43^ and dried at room temperature for 72 h until constant weight.

### SEM–EDX imaging

Scanning Electron Microscopy (SEM) images have been obtained on alive samples contaminated with a single spike of U solution in seawater at [U] = 10^−4^ M for 5 days.

To perform SEM and Energy Dispersive X-Ray spectroscopy (EDX) analyses, after contamination experiments, fresh samples of *A. nodosum* were rinsed with Milli-Q ultrapure water, and the various algal compartments were dissected and fixed in a 2.5% glutaraldehyde solution in 0.1 M sodium cacodylate buffer (pH 7.4) at room temperature for 1 h. Samples were then stored at 4 °C. After three rinsing cycles with Milli-Q ultrapure water, samples were dehydrated in a series of ethanol baths (70%, 96%, 100% three times, 15 min each). After a final bath in hexamethyldisilazane (HMDS, 5 min), samples were dried overnight. They were mounted on SEM stubs with carbon tape and coated with platinum (3 nm) prior to observations. SEM observations and EDX analyses were carried out with a Tescan Vega3 XMU scanning electron microscope (Tescan France, Fuveau, France) equipped with an Oxford X-MaxN 50 EDX detector (Oxford Instruments, Abingdon, U.K.) at an accelerating voltage of 20 kV.

### XAS

X-ray Absorption Spectroscopy (XAS) measurements were carried out on the MARS beamline of the SOLEIL synchrotron facility. MARS beamline is dedicated to the investigation of radioactive materials in the Hard X-ray range^44,45^. The optics of the line are composed by a water-cooled double-crystal monochromator that selects the incident energy of the X-ray beam and guarantees the horizontal focusing and two large water-cooled reflecting mirrors, used for high-energy rejection (the harmonic part) and vertical collimation and focusing. In this case, the monochromator was set with Si(220) crystals and the mirrors were set with Pt reflecting stripes at an angle of 2.7 mrad. All measurements were achieved in fluorescence mode using either a multi-element silicon drift detector (MIRION) or a multi-element high purity germanium detector (ORTEC). Data acquisition was performed at the U LII edge (20.95 keV) by performing energy calibration at the molybdenum K edge at 20 keV (the LIII edge could not be used because of the presence of Sr and Br in algae).

EXAFS and XANES spectra were both acquired on entire algal samples and on the different algal compartments. Solid pellets were prepared both with dried contaminated algae and by mixing the dry residue with polyethylene to obtain homogenous solid pellets. As polyethylene is only composed of light chemical elements, it does not interfere with the EXAFS/XANES measurements.

An alginate-U complex sample was prepared in vitro by mixing an alginate solution (1 g·L^−1^ of the extracted polysaccharides from 20 g of wet algal materials) with a uranyl nitrate solution (UO_2_(NO_3_).6H_2_O, [U] = 3.0 × 10^−3^ M). The obtained colloid was separated by the aqueous solution by centrifugation (10,000 *g*, 15 min). The EXAFS spectrum obtained for the uranyl-alginate complex was used as a reference for uranyl coordination in algal compartments.

XAS data was processed using the Athena and Artemis code of Demeter 0.9.25 package^46^ with E0 energy reference at the maximum of the absorption edge. The XAS spectrum was obtained by the subtraction of a linear pre-edge and post-edge background and a normalization. EXAFS data were adjusted using phases and amplitudes calculated with Feff7 code embedded in ARTEMIS code of Demeter 0.9.25 using two models: the meta-autunite phase and the uranyl acetate complex^46^.

µ-XRF cartography was also performed on *A. nodosum* receptacles and lateral branches. In order to reduce the beam-size. Additional Kirkpatrick–Baez (KB) mirrors were inserted in the beam. These consisted of rhodium coated trapezoidal mirrors (ZEISS) positioned at an angle of 2.5 mrad. The final beam-size was 24 × 27 µm2 (FWHM). Algal samples were cut by microtome saw (size 2 cm × 2 cm), fixed and embedded in Epon resin after dehydration. The imaging data were treated using PyMcA^47^ software. In addition, some micro-XAS measurements were performed on specific spots of the samples.

## Results and discussion

In the following section, a description of U accumulation following its uptake from seawater, its release and its distribution in *A. nodosum* tissues is presented. An initial assessment of the localization and molecular speciation of U in the algal compartments is proposed. No biological toxicity was investigated because it is beyond the scope of this work at this stage of the study.

### Accumulation and releasing studies

**Figure 2:**
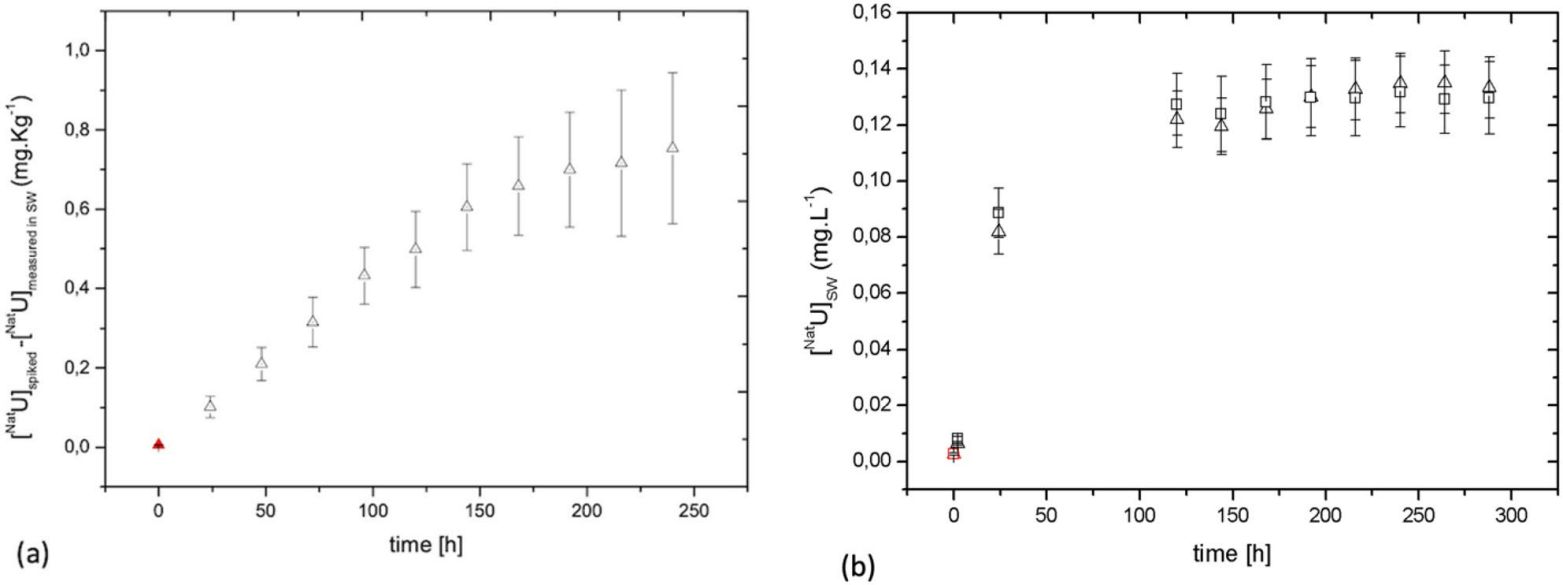
(**a**) Uptake curve of ^Nat^U in *A. nodosum*, calculated as ^Nat^U_spiked_—^Nat^U_measured in seawater_. (**b**) Eflux curves of contaminated *A. nodosum* during 12 days of observation. (△) aquarium 1 and (□) aquarium 2. The red points at time = 0 in Figures (**a**) and (**b**) correspond to the baseline concentration of U measured in natural seawater before spiking.

The uptake curves of ^Nat^U by *A. nodosum* were obtained using values calculated as the difference between the amount of U spiked in the aquarium and the amount of U measured in the aquarium 24 h after the spike (Fig. 2a and Fig. S1 of SI). Four *A. nodosum* individuals were contaminated in the same aquarium with a total uranium concentration of [U] = 10^–5^ M, corresponding to a single daily spike of 0.27 mg·L^−1^ per day (see contamination mode (a) in experimental details). Within the first 150 h a rapid uptake with a linear trend is observed, with a cumulative accumulation of almost 600 mg·Kg^−1^ of U. Such a trend may be linked to a biosorption mechanism, which is unrelated to an algal direct physiological response. This behavior was previously observed for the uptake of Cr(VI) by microalgae^48^.

At a later stage (from 150 to 250 h of contamination) the amount of accumulated U tends to stabilize. Arriving at such a steady state in the accumulation process may be related to the beginning of a depuration process occurring in *A. nodosum* that would partially reject the absorbed radionuclides, therefore reaching a balance between influx and efflux. It is known that the macromolecules (proteins, polysaccharides) play an important role in the depuration of tissues, allowing the algae to expel the non-essential metals^49^.

In order to confirm such depuration ability (efflux), and a balance between uptake and efflux, a releasing experiment was performed post contamination in 2 parallel aquaria with 3 individuals each and 4 L of fresh seawater (initial U concentration inside the algae in Aq1 [U] = 0.38 ± 0.02 mg. Kg^−1^ and in Aq2 [U] = 0.7 ± 0.1 mg. Kg^−1^). The releasing curve is presented in Fig. 2b. During the whole release experiment (300 h), the algae released between 20 and 30% of bioaccumulated U overall. The depuration process starts with a rapid release of U in the seawater and approximately after 5 days, a steady state is reached and a plateau is observed ([U] = 0.129 ± 0.001 mg·L^−1^, corresponding to a total amount of U = 0.5 mg). The passive sorption that we propose as the first accumulation mechanism of the U uptake might also explain the fast release observed in the first part of the efflux curve (0 to 125 h). Following this hypothesis, U that diffused inside the algal tissues in the intercellular matrix may easily be released into non-contaminated seawater^49,50^. In the second part of the releasing curve (125 to 300 h), several biological processes of detoxication can take place, which include chelation by intracellular ligands and cell deposit^51^. Such U-ligand interactions after the uptake were investigated and are reported below where first data on U in vivo speciation in *A. nodosum* are presented.

**Figure 3:**
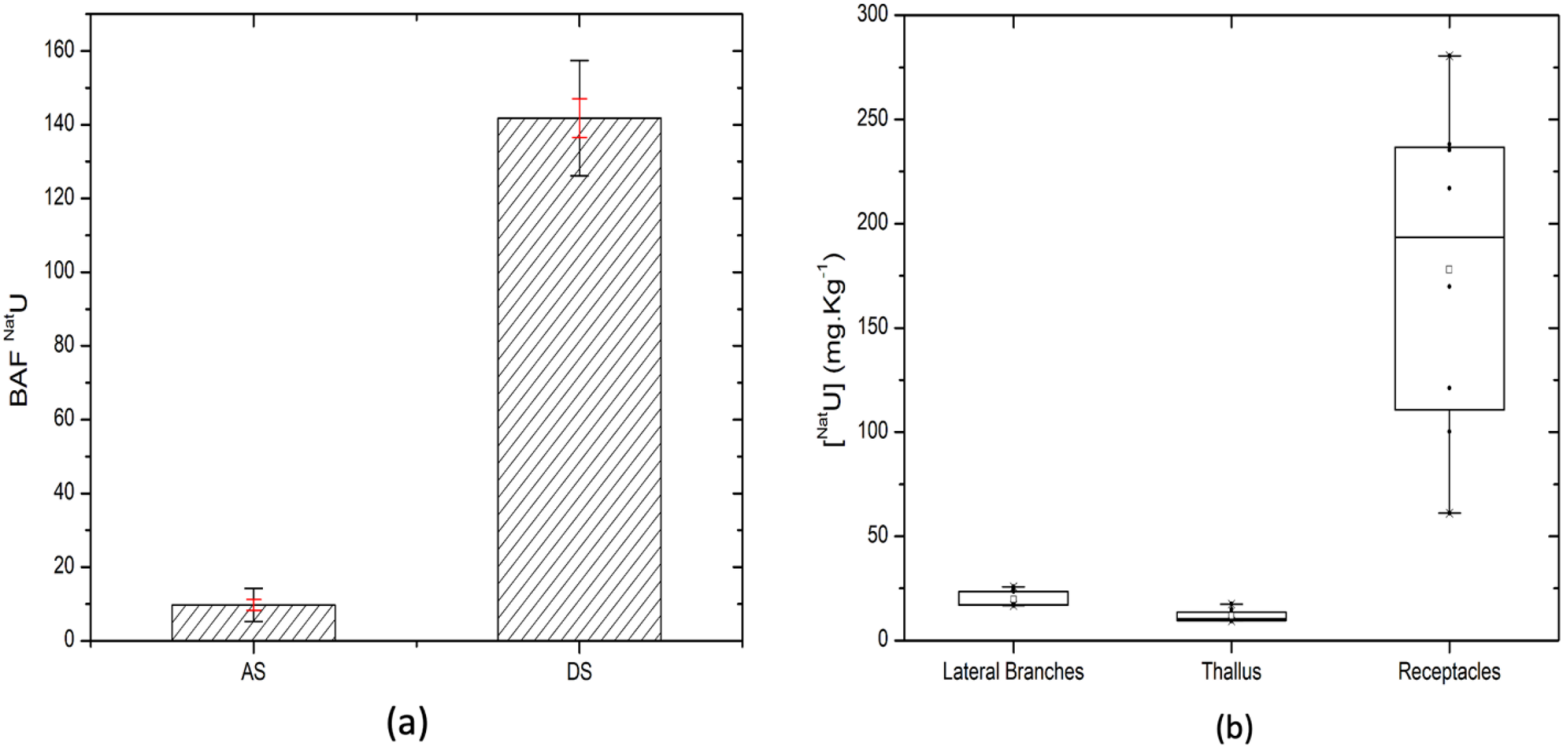
(**a**) BAF evaluated for 10 days uptake experiments for AS = alive algae (n = 9 individuals) and DS = dried algae (n = 9 individuals) (– dev. std and – mean error). (**b**) ^Nat^U concentration measured in *A. nodosum* separated compartments, calculated in mg·Kg^−1^ (dry wt.). Accumulation results statistically different (p < 0.01, independent student t-test^52^).

U has been further quantified in living *A. nodosum* individuals and in dried algal biomass. To this purpose, all the individuals were exposed to [U] = 10^–5^ M (one spike) in seawater for 10 days (see contamination mode (c) in experimental details). The Bioaccumulation Factor (BAF, defined in material and methods section) of *A. nodosum* was calculated. A control experiment was performed to determine the baseline concentration of U naturally present in algal tissues before contamination (equal to 0.27 ± 0.02 mg·Kg^-1^). Global BAF values are reported in Fig. 3a. Significant differences between the dried algal biomass samples (DS) and the live algal samples (AS) are observed. The dried biomass was able to uptake 90% more than the living organisms, which can be related to different accumulation mechanisms. Dried and dead algal biomass can only accumulate the ions through passive biosorption pathways, behaving like a biosorbent filter. Moreover, dried tissues don't have depuration processes: suppression of depuration ability might further explain the significant increase in the bioconcentration factor.

For the live individuals, the global BAF values have been complemented with specific values in the three compartments: receptacles, lateral branches and thallus. Data are reported in Fig. 3b. The U amount follows in the order (with partial BAF values): receptacles (49 ± 12) >  > lateral branches (5.2 ± 0.7) >  = thallus (3.1 ± 0.5). This significant discrepancy between the receptacles and the other organs may be due to different uptake mechanisms. Table S1 of SI summarizes the values of partial BAF data and accumulated concentrations in each algal compartment.

The above data suggest that in living algae, the uptake process may result from two different mechanisms: active uptake on the one hand and sorption, as in the dead tissues, on the other hand. For the uptake, the receptacles (containing the reproductive organs) are the main targets. Note that U accumulation experiments with live *A. nodosum* compartments were performed in April–May during the reproductive season (spring/summer), meaning that the annual receptacles were present and in mature stages (they usually disappear after the reproductive season)^34^.

### Uranium localization

**Figure 4:**
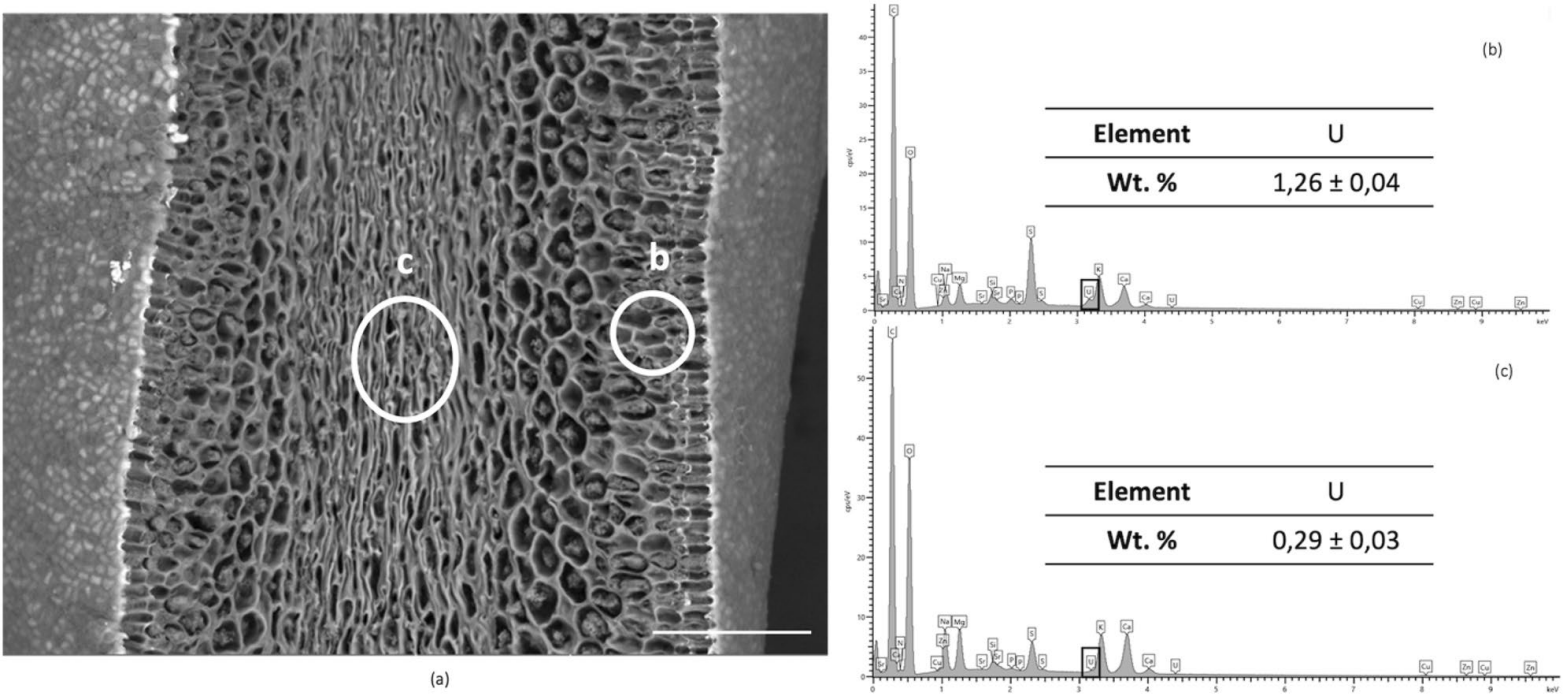
(**a**) Scanning electron micrographs (SEM) of dehydrated tissues from contaminated lateral branch of *A. nodosum*, showing the surface and inner meristoderm structures. EDX spectra obtained from (**b**) algal surface tissues (zone (**b**)) and (**c**) inner part of tissues (zone (**c**)). Scale bar 100 µm.

In order to further characterize the uptake mechanisms, U localization within contaminated *A. nodosum* individuals was investigated using a combination of Scanning Electron Microscopy (SEM–EDX) and micro–X-ray Fluorescence Spectroscopy (μ-XRF). Two algal compartments were further analyzed after contamination: receptacles and lateral branches (corresponding to both compartments accumulating the most and at values higher than the EDX detection limit). Lateral branches present a superficial distribution of U, that penetrates just a few layers of primary cells (Fig. 4). An external bright field, not visible in non-contaminated algae (Fig. S2 of SI) suggests that U is mainly located in the extracellular matrix. The elemental EDX spectra, Fig. 4b and c, of two different sections of the branch confirm the presence of U with an increasing amount in the superficial tissues. The competition between U and other cations has not been considered here, but it is worth bearing in mind that experiments have been conducted in natural seawater, where essential metal cations (Ca^2+^, Mg^2+^ or Zn^2+^) play an important role in the biomass survival^53–55^. Therefore, accumulation of U on the algal surface may interfere with the uptake of such essential cations, as they probably share the same channels of absorption but this is beyond the scope of the present study at this time.

Alginic acid, with its carboxylate residues, is known to be involved in metal chelation and ion exchange in algal tissues^56^. Among all the other cell wall polymers, it shows a high affinity for divalent cations, such as formally the uranyl oxocation^7^. In addition, alginic acid is known to be present together with cellulose, in the cell wall structure^7^, where U has been mostly accumulated. This leads to the hypothesis that in *A. nodosum* lateral branches, the principal path of the absorption of U would be related to the presence of polysaccharides, with a focus on the alginic acid polymers.

**Figure 5:**
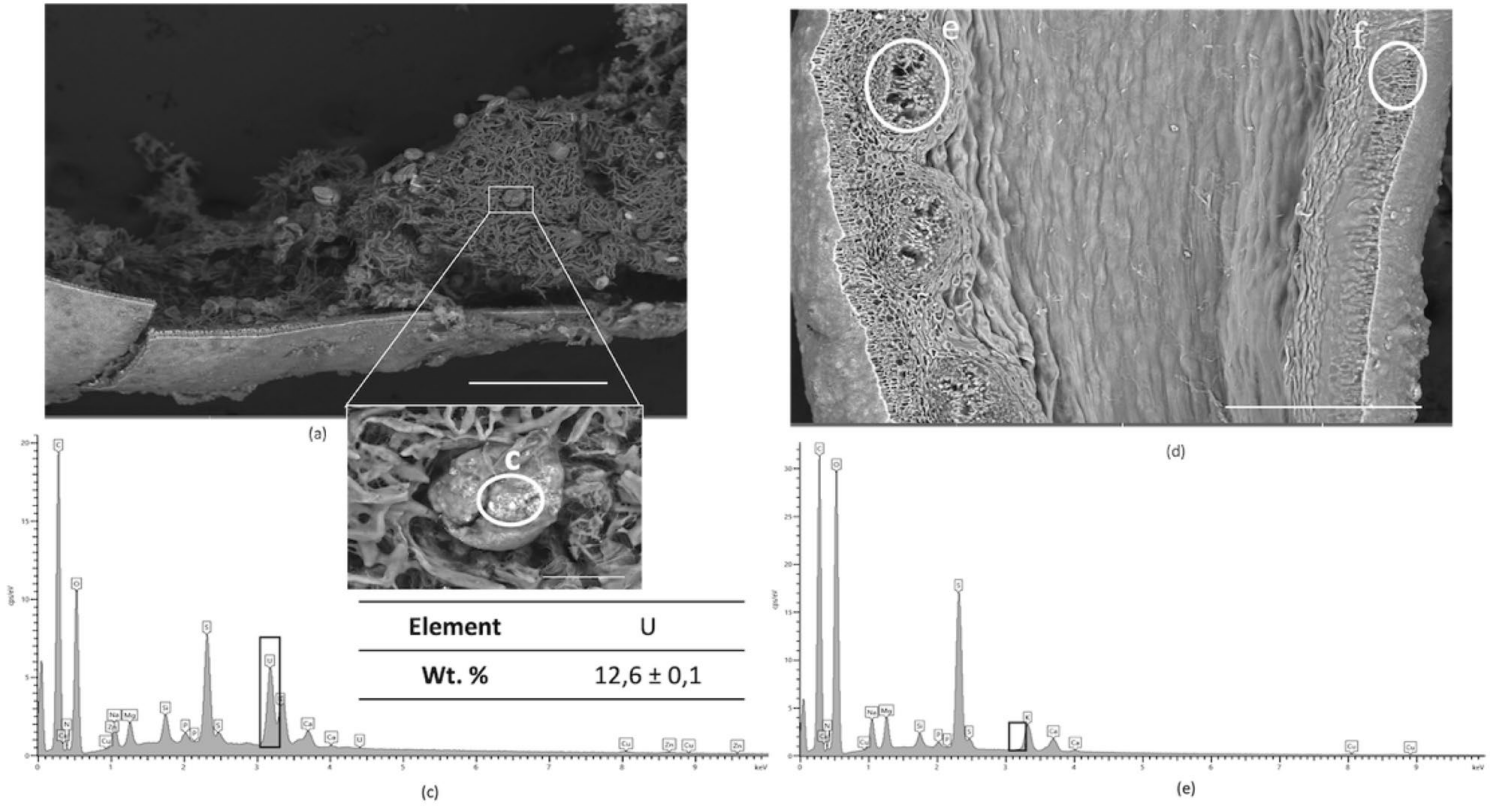
SEM images of contaminated receptacles from 2 different individuals of *A*. *nodosum* exposed for 5 days at 10^–4^ M of uranyl nitrate and the EDX spectra. (**a**) Micrograph of a female receptacle, blown up after dehydration, showing the exposed *oogonium* (**b**) Zoom on the *oogonium* structure, filled with eggs contaminated with ^Nat^U, stored under the cell membrane (translucent white spots). (**c**) EDX spectrum obtained in the middle of one egg highlighting the presence of accumulated uranium (zone (**c**)). (**d**) Cross section SEM image of male receptacle showing the *antherridium* filament structure. (**e**) EDX spectrum recorded inside the male conceptacle, zone **e**, showing no visible U. Scale bar: a = 500 µm, b = 50 µm and d = 500 µm.

Figure 5 shows the SEM images and the EDX elemental spectra of two receptacles (a female and a male) from two different individuals of *A. nodosum*. This dioecious alga^34^ carries either male or female reproductive organs inside the receptacle’s compartments on two distinct unisexual individual. Each female or male receptacle contains conceptacles where the female or male gametes are enclosed, respectively the oogonium, filled with eggs (female), and with the antheridium filaments, containing the spermatozoids (male). Oogonium eggs are visible in Fig. 5a,b, corresponding to a receptacle from a female individual in the maturation stage, where several conceptacles were empty, after releasing the oogonium spheres. The elemental EDX spectrum (Fig. 5c) reveals the presence of U inside the eggs. In this location, the concentration of U is higher than that measured by EDX in the lateral branch, also in agreement with the BAF values obtained with the uptake experiments (Fig. 3).

The second analyzed receptacle (Fig. 5d) belongs to a male specimen: an oval shaped structure is visible containing fibrous cells, attributable to a male conceptacle with fertile antheridia filaments. The EDX spectra show the absence of U inside of the antheridia filaments (Fig. 5e). Instead, it is mainly distributed at the surface of the (male) receptacle visible as a white deposit in Fig. 5d (EDX zone f represented in Fig. S3 of SI).

There is, therefore, a clear difference between the accumulation mechanisms of both female and male conceptacles. These data underline the necessity to further investigate the U speciation in the two different algal compartments, lateral branches and receptacles (male and female conceptacles). Also, the significant differences between the U concentrations in *A. nodosum* compartments call for differences in speciation. Finally, differences between male and female conceptacles may be expected, as different spatial fingerprints are oberved with SEM imaging.

### Uranium speciation

As a first attempt to assess U speciation after uptake, X ray Absorption Spectroscopy (XAS) spectra and µ-X ray Fluorescence (µ-XRF) cartography in different algal comparts have been recorded at the LII edge of U. All the µ-XRF images were obtained from different individuals contaminated with [U] = 10^–5^ M (1 spike, 10 days), see contamination mode (a) in experimental details.

The U accumulation in lateral branches and thallus is very low, as already explained above. These tissues indeed showed an average U concentration of ca. 12 and 19 ppm, respectively. These values are at the limit of the estimated XAS detection limit under our experimental conditions. As both algal tissues (lateral branch and thallus) have the same meristoderm structures, only a lateral branch sample (with slightly higher U content compared to thallus) was analyzed. In agreement with the results visualized in the SEM images of Fig. 4a, the cartography of the lateral branch (Fig. S4 of SI) shows a diffuse distribution of U. Note that intensities on all the µ-XRF images are qualitative since no internal calibration of the U fluorescence intensity could be performed. This diffuse distribution is in agreement with the observations using the SEM images, underlying an absorption phenomenon.

**Figure 6:**
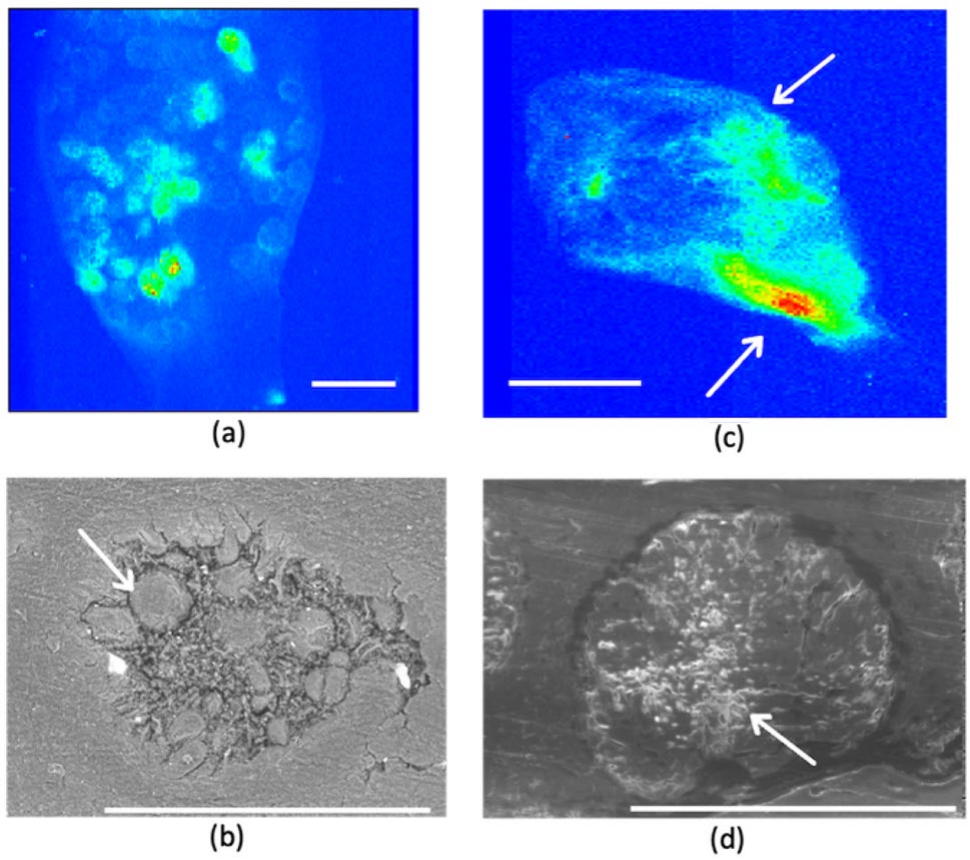
(**a**) µ-XRF cartography of uranium distribution (L_II_ edge) inside a female receptacle of *A. nodosum*. Uranium hotspots are visible inside the conceptacles (red marks). (**b**) SEM image of the female conceptacle from the receptacle of Fig. 6a, arrows point to the oogonium cells (**c**) µ-XRF cartography of uranium distribution (L_II_ edge) inside a male receptacle of *A. nodosum*. (**d**) SEM image of a male conceptacle from the receptacle of Figure (**c**), arrows point to the antheridium filamentous structure cells, typical of male gametes. Scale bar: a = 1.6 cm, b and d = 500 µm, c = 2 cm.

Concerning the receptacle compartments, two samples belonging to a female and a male respectively were analyzed (Fig. 6), underlining distinct U spatial distribution as already observed with the SEM–EDX images. Within the female receptacle (Fig. 6a), the highest amount of uranium has been found inside the conceptacles, in agreement with the distribution revealed by SEM–EDX (Fig. 5a). For comparison, female gametes are also identified by SEM observations at a smaller scale (500 µm, Figs. 6b).

The µ-XRF cartography of the male receptacle (Fig. 6c) only shows adsorbed U on the surface. Figure 6d shows an example of the male conceptacle, for comparison, at a smaller scale (500 µm). No comparison is possible between the two cartographies because of the lack of internal normalization (the color scale is arbitrary and independent between the images).

**Figure. 7:**
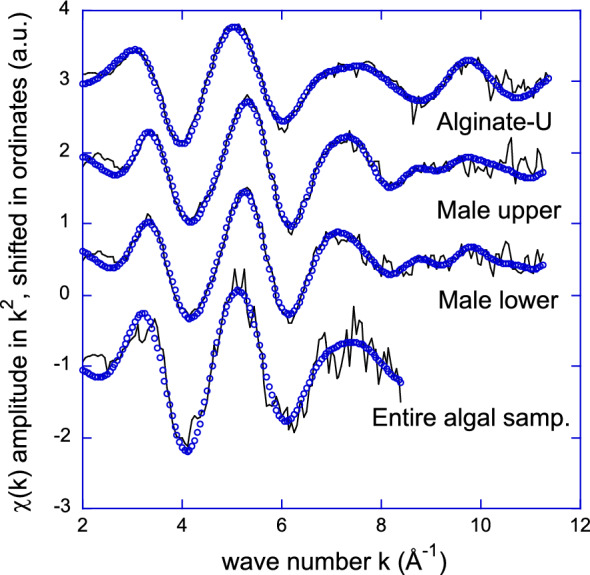
Experimental (straight line) and adjusted (dots) EXAFS spectra of Alginate-U (bulk EXAFS), *A. nodosum* male receptacle upper part (imaging EXAFS), *A. nodosum* male receptacle lower part (imaging EXAFS) and *A. nodosum* entire algal sample (bulk EXAFS).

EXAFS spectra recorded on two different U spots within the male receptacle (one lower and one upper, pointed out by the arrows in Fig. 6c) are compared in Fig. 7. Both spectra are qualitatively similar. Best fit parameters reported in Table 1 have been obtained using an uranyl phosphate phase (the meta-Autunite phase^9^) as a model. Agreement between the m-Autunite phase (crystallographic averaged distances: 2O_ax_ at 1.77 Å, 4 O_eq_ at 2.28 Å and 4 P_monodentate_ at 3.60 Å^57^) and the experimental fitted data is noteworthy, suggesting that in both parts of the male receptacle, U is incorporated as a phosphate phase, namely a pseudo Autunite phase. The equatorial coordination number, *ca*. 5 O_eq_, does not differ significantly within the error from the inorganic m-Autunite solid structure (4 O_eq_). Values of Table 1 are also in very good agreement with the literature data obtained from U speciation in bacteria^58^. Moreover, the average distance of U-O_eq_ for U species formed by the bacteria *Acidithiobacillus ferroxidans* is 2.36 Å, very close to the EXAFS value found in this study (2.32 – 2.35 Å). For both EXAFS spectra the U…P distance of 3.61 -2.65 Å is typical of a monodentate phosphate group within the error^58,59^.

**Table 1:** EXAFS data on different uranium (VI) complexes in comparison with the spectroscopic data of the m-Autunite solid state structure.

	Oxo contribution	Second contribution	Third contribution
Alginate-U	*2* O at 1.80 (1) Å	6.3 (9) O at 2.42 (2) Å	–
	${\sigma }^{2}$= 0.0023 Å^2^	${\sigma }^{2}$= 0.0091 Å^2^	
	S_0_^2^ = 1.0, e_0_ = 4.0 eV, Q = 70, R = 0.9%		
Male receptacle upper part	*2* O at 1.81 (1) Å	5.3 (8) O at 2.33 (2) Å	*4* P at 3.65 (4) Å
	${\sigma }^{2}$= 0.0043 Å^2^	${\sigma }^{2}$= 0.0096 Å^2^	${\sigma }^{2}$= 0.0108 Å^2^
	S_0_^2^ = 1.0, e_0_ = 3.6 eV, Q = 26, R = 1.0%		
Male receptacle lower part	*2* O at 1.82 (1) Å	5.5 (10) O at 2.35 (2) Å	*4* P at 3.61 (7) Å
	${\sigma }^{2}$= 0.0048 Å^2^	${\sigma }^{2}$= 0.0097 Å^2^	${\sigma }^{2}$= 0.0074 Å^2^
	S_0_^2^ = 1.0, e_0_ = 3.5 eV, Q = 14, R = 1.7%		
Entire algal sample	*2* O at 1.81 (1) Å	8.0 (46)* O at 2.40 (3) Å ${\sigma }^{2}$ = 0.0155 Å^2^	
	${\sigma }^{2}$= 0.0016 Å^2^		
	S_0_^2^ = 1.1, e_0_ = 7.5 eV, Q = 15, R = 2.1%		

*S*_*0*_^*2*^ is global amplitude factor, e_0_ is the energy threshold, Q is the quality factor of the fit and r (%) is the agreement factor with the fitting. ${\sigma }^{2}$ is the Debye Waller factor of the considered scattering pass. Numbers in bracket correspond to the estimate uncertainties of the last digit. Numbers in italics have been fixed.

*Due to the low signal to noise ratio and short k range of this spectrum, the uncertainty on global amplitude (*ie.* N x S_0_^2^) is close to 50%.

Unfortunately, no EXAFS spectrum with an adequate signal to noise ratio could be recorded on the oogonium female conceptacle for comparison. We attribute this failure to the smaller size of the U spots in the oogonium, as can be seen in Fig. 6a, at the lower limit of the beam size spot used for EXAFS recording (24 × 27 µm^2^, FWHM).

Among all the cellular compounds, alginate is the major component of brown algal cell walls, as already mentioned. It represents 40% of algal dry weight^60^. It is composed by mannuronic (M) and guluronic (G) acid monomers^61^ that are able to coordinate divalent cations, undergoing an ionic crosslinking between G-residues of adjacent polymeric chains^7^. As a polymeric acid with strong affinity for metallic cations^62^ and its high concentration in brown algal tissue, it is a good candidate for U complexation in the tissues. The complex formed between alginate extracted from *A. nodosum* and U (see experimental section) has been used as a comparative model for XAS measurements. The best fitted parameters of the corresponding EXAFS spectrum of alginate-U complex are presented in Table 1. The first shell corresponds to both of the typical axial oxygen atoms while the second one is composed of 6.3(9) O atoms at 2.42(2) Å. There is no improvement of the fit by adding a third shell of carbon atoms, for instance. Inclusion of a U–O–C multiple scattering path as in monodentate carboxylates also did not lead to any improvement. This suggests that in the alginate-U model complex, the form of the uranyl oxocation resembles its aquo form or a mix of aquo and carboxylate forms. The carboxylate (from alginate) complex is probably disordered enough that any C contributions is meaningless for the EXAFS probe. For comparison, a XAS spectrum of the lateral branches, where alginate is abundant was recorded in μ-XRF mode from spot g of Fig. S4 in SI. Unfortunately, the U content was too low to record an EXAFS spectrum with a workable signal to noise ratio. A XANES spectrum was recorded instead and it is compared to the XANES spectra of alginate-U complex and of meta-autunite mineral phase (derivative spectra are presented in Fig. S5 of SI). Although there is a slight difference between the autunite and alginate-U models, the signal to noise ratio of the lateral branches XANES precludes any further interpretation of the U speciation in this compartment.

Lastly, the EXAFS spectrum of the entire *A. nodosum* sample with a U concentration of 24 ± 1 (mg. Kg^−1^) (see experimental section) was also recorded. Best fit parameters presented in Table 1 reveal a close similarity with the alginate-U complex, although this interpretation is very limited by the very low signal to noise ratio of the spectrum (very high uncertainties in the fitted parameters) and short k range (Fig. 7). It must also be kept in mind that only average U speciation will be obtained with this measurement. For instance, the speciation obtained on the hot spots of the male conceptacle is averaged with the speciation over the entire tissue. It can be concluded that this measurement only suggests that the U speciation over the entire algae is not a pure pseudo autunite phase, as it is specifically in the male conceptacles. Although this is not a direct proof, it backs the hypothesis that alginate may play a specific role in the complexation of U in the lateral branch tissues. More investigation is needed to improve the mapping of U speciation in the different compartments of *A. nodosum*.

## Conclusion

This study investigates the bioaccumulation of natural uranium by the brown alga *Ascophyllum nodosum* as well as the distribution and the chemical speciation of this radioelement inside the algal tissues. Individuals of *A. nodosum* were exposed to a defined contamination dose in order to mimic a seawater pollution event.

The ability of this brown algal species to bioaccumulate U was observed and quantified. The obtained results show an accumulation in the first 150 h with an average U(VI) amount of 600 mg⋅Kg^−1^ reaching a steady state in the final part of the contamination period. A different uptake mechanism for live specimens and dried tissues has been proposed: an active bioaccumulation, followed by a depuration process for the former, and a passive biosorption mechanism for the latter. The ability of live algae to purify and detoxify their tissues justifies the lower bioaccumulation factor found, with an average of 10 ± 2, which is much lower compared to the dry biomass. In fact, the dry biomass, lacking this ability, shows a continuous uptake along the 10 days of contamination, with a bioaccumulation factor 90% higher (142 ± 5). The quantification of the total U inside the different tissue types revealed the presence of an internal compartmentalization, with higher concentrations within the reproductive organs (the receptacles) compared to vegetative parts. A significant difference was found in the U concentration of the three algal parts investigated: 49 ± 12 for the receptacles, 5.2 ± 0.7 for the lateral branch and 3.1 ± 0.5 for the thallus.

The combination of complementary imaging techniques (SEM–EDX and µ-XRF) showed different U localization inside the algal tissues, with a widespread surface distribution in the main tissues, such as thallus and lateral branch together with the male receptacles and several hotspots inside the oogonium eggs of the female receptacles. The speciation analysis performed on the male gametes revealed the presence of a pseudo autunite phosphate phase, showing an average of 5 O_*eq*_ complexing the uranyl ion in the second shell at 2,3 Å and 4 monodentate P ligands at 3.6 Å in the third shell. Unfortunately, comparison with the female gametes was impossible because of the very low signal to noise ratio. Similarly, XAS measurements on the lateral branches (XANES) were not conclusive with regards to the occurrence of an alginate-U complex in these tissues. Finally, the EXAFS spectrum of the entire algae resembles that of the alginate-U complex, suggesting indirectly that the average speciation is not a pure pseudo autunite phase as it is specifically in the male conceptacles. Although this is not a direct proof, it backs the hypothesis that alginate may play a role in the speciation of U except in the male conceptacles.

The integration of complementary techniques (ICP-MS, μ-XRF, EXAFS, and SEM) yields new insights into both the accumulation and spatial distribution of uranium across different algal sections. This holistic methodology sheds light on the mechanisms of uranium bioaccumulation and how uranium is compartmentalized within the macroalgae. These first findings provide new evidence to understand and quantify the biochemical consequences of uranium contamination and transfer within marine species. Also, they provide useful information for the development of environmental monitoring and remediation strategies.

Although partially limited because of the difficulty to obtain usable XAS spectra with an adequate signal to noise ratio, these results open up new perspectives on the characterization of the algal macromolecules involved in the uranium uptake. This is a first step to decipher the chemical mechanisms of the uptake, and to highlight metabolic differences between female and male gametes involved in the U bioaccumulation mechanism.

### Supplementary Information

Supplementary Information.

## Data Availability

The datasets used and/or analysed during the current study are available from the corresponding author on reasonable request.
