# Supplementary material for: Exploring uranium bioaccumulation in the brown alga Ascophyllum nodosum: insights from multi-scale spectroscopy and imaging
Source: Sci Rep. 2024 Jan 10;14:1021. doi: 10.1038/s41598-023-49293-w (PMC10781969; doi:10.1038/s41598-023-49293-w)
Supplement: Supplementary file 1 — Supplementary Information. [file 41598_2023_49293_MOESM1_ESM.docx]

**Supporting Information file**

**Exploring uranium bioaccumulation in the brown alga Ascophyllum nodosum: insights from multi-scale spectroscopy and imaging**

Micol Zerbini^1^, Pier Lorenzo Solari^2^, Francois Orange^3^, Aurélie Jeanson^1^, Catherine Leblanc^4^, Myriam Gomari^1^, Christophe Den Auwer^1^, Maria Rosa Beccia^1^*

*^1^ Université Côte d’Azur, CNRS, Institut de Chimie de Nice, UMR 7272, 06108, France*

*^2^ Synchrotron SOLEIL, L’Orme des Merisiers, Départementale 128, F-91190 Saint-Aubin, France*

*^3^ Université Côte d’Azur, Centre Commun de Microscopie Appliquée, 06108, France*

*^4^ Sorbonne Université, CNRS, Station Biologique de Roscoff, UMR 8227, 29680, France*

maria-rosa.beccia@univ-cotedazur.fr





**Figure S1**: [^Nat^U] concentration inside the aquarium during the contamination of 4 individuals of A. nodosum in 10 days of observation. (▽) daily spiked U and (□) daily measured U in sewater after 24h from the spike.

**Table S1:** ^Na^U concentration (mg·Kg^-1^ – dry weight of biomass) and Bioaccumulation Factor of the A. nodosum algae. compartments.

| Algae compartment | BAF | [U] (mg Kg^-1^) |
| --- | --- | --- |
| *Receptacle* | 49 ± 12 | 178 ± 27 |
| *Lateral branch* | 5.2 ± 0.7 | 19 ± 1 |
| *Thallus* | 3.1 ± 0.5 | 11.7 ± 0.9 |


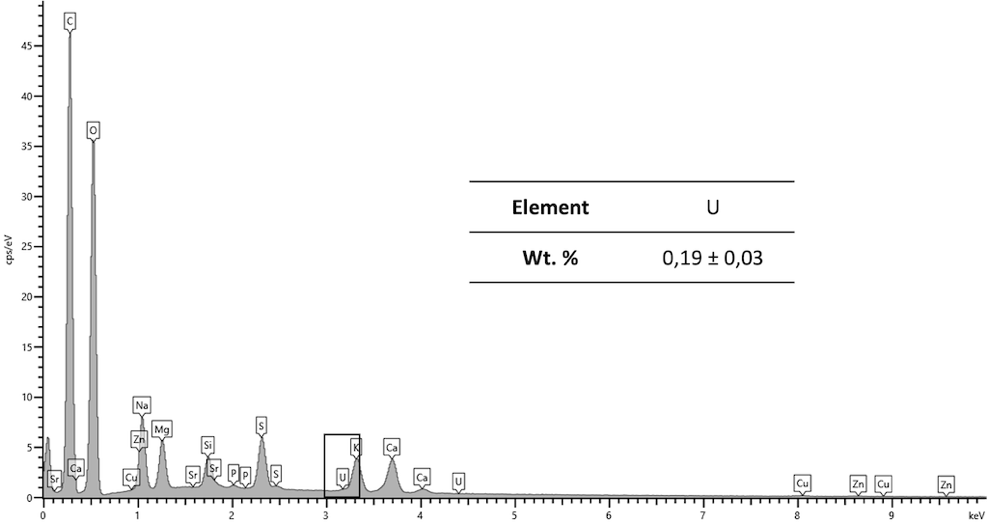


**Figure S3:** EDX spectrum obtain on the surface of the A. nodosum male receptacle reported in Figure 5d, showing the presence of U (wt% 0.19 ± 0.03)


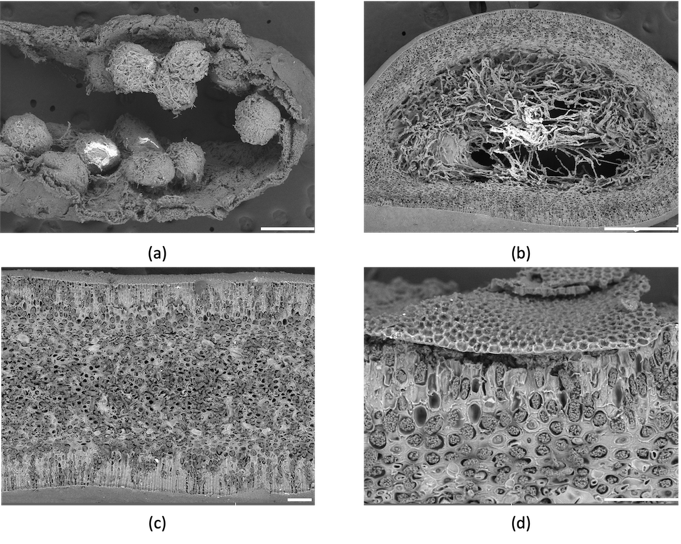


**Figure S2**: SEM images on non-contaminated tissue of A. nodosum. a) Receptacle with visible conceptacles, b) inner part of a floating air bladders, c) lateral branch and d) thallus. Scale bar (a)-(b) =500 µm and (c)-(d) = 100 µm.


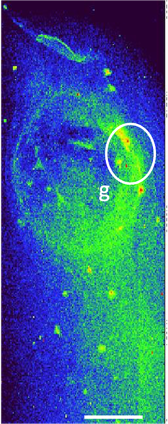


**Figure S4**: µ-XRF cartography of uranium distribution inside a lateral branch sample from *A. nodosum*, with a widespread presence of U at low concentration (green) and several hotspots (red). Scale bar = 1 cm.

**Figure S5**: Derivative XANES spectra at the U L_II_ and L_III_ edges, normalized in energy for comparison, of the meta-Autunite model (blue), of the alginate-U reference complex (green) and of the lateral branch (red) of A. nodosum thallus.
